# Malaria control in potable water and in biodiversity rich habitats: need and opportunities for biological control agents

**DOI:** 10.1186/1475-2875-11-S1-P31

**Published:** 2012-10-15

**Authors:** Sevvandi Javakodv, GK Achini Wathsala Fernando, WM Hiranya Kelum Wijenayake, RD Jeevani Harishchandra, WM Tikiri Banda Wanninayake, JM Pushpa Kumar Jayasinghe, Managala Yatawara, PK Nirosha Chandani Liyanage, Sarath L Deniyage, Gavvrie NL Galappaththy, S Ravindra Jayanetti, M Devika B Perera

**Affiliations:** 1 Department of Aguaculture and Fisheries, Wayamba Univesity of Sri Lanka, Makandura, Gonawila, Sri Lanka; 2 University of Kelaniya, Dalugama, Sri Lanka; 3 Anti-Malaria Campaign, Colombo 05, Sri Lanka; 4 Anti Malaria Campaign, Anuradhapura, Sri Lanka; 5 Anti Malaria Campaign, Kurunegala, Sri Lanka

## Background

During recent years, the contribution of man-made aquatic/semiaquatic habitats for breeding of malaria vector *Anopheles culicifacies* has increased, challenging the efforts of eliminating indigenous malaria from Sri Lanka. Some serve as important aquatic habitats with rich biodiversity while others are constructed to extract potable water and for agriculture in water sparse dry zone. These restrictions call for alternative control measures. A study explored the presence of malaria larvae and possible use of an indigenous fish species as a part of an on -going attempt for alternative malaria control methods.

## Materials and methods

Three types of potential mosquito breeding habitats; abandoned clay pits and quarry pits from wet (rain fall (RF) >2500mm ) and intermediate ( RF = 1750-2500mm ) zones and agricultural wells in dry zone (RF< 1750mm) were mapped and were processed with MapSource^®^ software. Anophiline larvae in clay pits (n = 41) and quarry pits (n = 39) were sampled taking six dips at each point at 10m intervals of the perimeter using a standard 350 ml dipper. In agricultural wells (n = 43 in Wagollakada and n = 89 in Rathmale regions of dry zone), were sampled with a 2L dipper at four points. Third and fourth instars of larvae were identified to the species level and percentage occurrence of larvae and larval density were calculated. Simultaneously, *Aplocheilus parvus*, a common surface dwelling predatory indigenous fish species available in both lotic and lentic systems in all zones of Sri Lanka , was collected every 2 hours for 24 hours to determine its preference for mosquito larvae.

## Results

The results indicated that 62% of clay pits and 76% of quarry pits were positive for Anopheline larvae while 31 % and 37% of agricultural wells contained Anopheline larvae in Rathmale and Wagollakada respectively. Malaria vector *An. culicifacies* was present in 3% of clay pits at a density of 0.001 larvae/dip and 24% of quarry pits at the density of 0.027 larvae/dip. Among agricultural wells, 11% was positive for *An. culicifacies* in Rathmale at 0.097 larvae/dip whereas 11.63% wells in Wagollakada had a density of 0.106 larvae/dip. Moreover, potential malaria vectors *An. varuna*, *An. vagus* and *An. jamesii were* also recorded from all three types of habitats. Agricultural wells in Rathmale were also the habitat for Culicines at 0.899 larvae/dip including vector of Japanese Encephalitis *Culex tritaeniorhynchus.* According to the diet composition analysis, *A. parvus* diet mainly consisted of adult or larval stages of class Insecta (Coleoptera, Hymenoptera and other unidentified insect parts and insect larvae) and class Maxillopoda (Copepoda). A time sex interaction explained the gut fullness with males having a peak gut fullness during 1630 hours whilst females had the peak gut fullness at 1230 hours (p < 0.001). Results suggested that insect parts and Coleopterans were the dominant food items present in all time periods (p < 0.001).

## Conclusions

The need for alternative malaria control measures is highlighted. Currently *A. parvus* and guppy (*Poecilia reticulata*) are being experimentally introduced to above habitats and larval densities are being monitored whist the sensitivity of *A. parvus* to commonly used agricultural chemicals are being tested.

